# A comparative analysis of link removal strategies in real complex weighted networks

**DOI:** 10.1038/s41598-020-60298-7

**Published:** 2020-03-03

**Authors:** M. Bellingeri, D. Bevacqua, F. Scotognella, R. Alfieri, D. Cassi

**Affiliations:** 1 Dipartimento di Fisica, Università di Parma, via G.P. Usberti, 7/a, 43124 Parma, Italy; 2 PSH, UR 1115, INRA, 84000 Avignon, France; 3 Dipartimento di Fisica, Politecnico di Milano, Piazza Leonardo da Vinci 32, 20133 Milano, Italy; 4 Center for Nano Science and Technology@PoliMi, Istituto Italiano di Tecnologia, Via Giovanni Pascoli, 70/3, 20133 Milan, Italy

**Keywords:** Statistical physics, thermodynamics and nonlinear dynamics, Computational biology and bioinformatics, Computational science

## Abstract

In this report we offer the widest comparison of links removal (attack) strategies efficacy in impairing the robustness of six real-world complex weighted networks. We test eleven different link removal strategies by computing their impact on network robustness by means of using three different measures, i.e. the largest connected cluster (*LCC*), the efficiency (*Eff*) and the total flow (*TF*). We find that, in most of cases, the removal strategy based on the binary betweenness centrality of the links is the most efficient to disrupt the *LCC*. The link removal strategies based on binary-topological network features are less efficient in decreasing the weighted measures of the network robustness (e.g. *Eff* and *TF*). Removing highest weight links first is the best strategy to decrease the efficiency (*Eff*) in most of the networks. Last, we found that the removal of a very small fraction of links connecting higher strength nodes or of highest weight does not affect the *LCC* but it determines a rapid collapse of the network efficiency *Eff* and the total flow *TF*. This last outcome raises the importance of both to adopt weighted measures of network robustness and to focus the analyses on network response to few link removals.

## Introduction

Understanding how the removal of nodes or links affects the functioning of a network is a major topic in science^1–6^. It permits to rank nodes (or links) according to the consequence of their removal on the system. Also, it provides information for increasing the robustness (resilience) of networked systems^7,8^. In fact, once the most important nodes-links are found, one can increase the network robustness by protecting these key components, for example by directing resources to preserve important internet routers or implementing policies to secure most important bridges (or roads) in transportation networks. For these reasons, many studies analysed the effect of removal (attack) strategies on real-world complex networks in different fields of science^1,2,9–17^.

Yet, recent classic outcomes indicated that many real-world complex networks showed ‘robust yet fragile’ nature, i.e. they are robust to the random removal of nodes but very fragile to the attack of the most connected node components^1,13,18,19^. Following these outcomes, a plethora of attack strategies have been proposed to determine the sequence of nodes removal that maximise the damage in the networks^5,6,12,20–22^. Most of these analyses consist in measuring the decrease in some indicators of the network integrity (functioning) following empirical removal of nodes-links^4–6,12,15,20–22^.

### The link removal strategies

The main idea of link removal (also called link attack, link pruning or edge attack) strategies can be traced back to the Granovetter “The Strength of Weak Ties”^23^ paper, that arguably contains the most influential sociological theory of networks. In this classic analysis the social interpersonal relationships were categorized in strong, weak or absent. A strong tie (link) is that one linking someone within a close circle of family and friends. Strong ties are essential for real communities but they typically tie together groups with a great deal of similarity. Thus, there are more tenuous connections to carry new information and perspectives to their groups. Granovetter central argument is that contacts maintained through weak ties are more likely to be bridges to socially distant network people communities, which provide access to novel information and resources fundamental for system functioning^23^. The classic weak-strong ties classification adopted for social networks has been translated outside of the social networks theory^3^. In most real world networks, a gradation of interactions exists, usually quantified by the link (ties) weight, which reflects important functioning features such as e.g. capacity in transportation routes and communication networks, the number of synapses between neurons, the strength of a prey-predator relationship in ecological networks, or memories reinforced in brain networks. Further, in many of these real-world systems the difference in the weight of the link can span several orders of magnitude, with many links of small weight (weak link) and a small fraction of links of very high magnitude (strong links)^3^.

Following the Granovetter main idea, in recent year link removal analyses conducted over economic complex systems showed that weak connections support the overall connectivity of the network significantly more than the strong links^24^. Similar counterintuitive vulnerability of the network connectivity to weak links removal was found in social networks of human interactions from mobile phone call record^25,26^ and it was then reproduced in models of complex weighted networks^27,28^. These analyses outlined the importance of weak links in sustaining the functioning of real-world networks^29^.

With the aim to clarify the role of weak and strong links, Pajevic and Plenz^3^ classified real-world networks in two main categories i.e. integrative and dispersive networks. Integrative networks showed the local link weight organization in which strong links preferentially occur between nodes with overlapping neighborhoods (e.g. strong links occur between nodes belonging to the same community); on the contrary dispersive networks presented strong links preferentially joining nodes with non-overlapping neighbors (e.g. strong links occur between different communities). The different embedding of the strong links affects network vulnerability, e.g. the clustering coefficient of the integrative networks is highly vulnerable to the removal of strong links and robust to weak removal, on the contrary the clustering of dispersive networks rapidly decreases with weak links removal^3^. Recently, Bellingeri *et al*.^30^ analyzed the response of real-world weighted complex networks to link removals showing how higher level of link weights heterogeneity may enhance the vulnerability of these real-world systems. Further, they found a sharp decrease of the network efficiency (*Eff*) under the removal of links with higher weight, revising the role of strong links and raising the importance to perform methodologies considering the heterogeneity in link weights in the real-world networks^30^.

**Figure 1:**
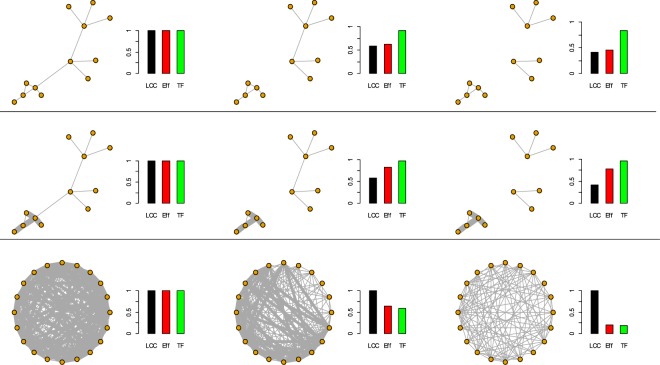
The network functioning measures. Simple examples of model networks under link removal depicting the different interpretation of the system functioning furnished by the measures used in this paper. The bar plot at the right of each network indicates the value of the functioning measures (normalized on the initial network functioning value). The links width indicates the link weights. Top row: topological (binary) sparse network; half row: weighted sparse network; bottom row: fully connected weighted network. The *LCC* quickly collapses in the sparse topological (binary) network with two link removals; *Eff* follows the *LCC* decrease whereas the *TF* holds almost unaltered (Fig. 1 top row). Introducing heterogeneity in link weights over the same sparse network, now the *Eff* does not follow the *LCC* decrease acting more similar to the *TF* (Fig. 1 half row). In the last row we depict a fully connected weighted network under higher weight link removals (strong links) the *LCC* holds constant under strong links pruning where instead *Eff* and *TF* quickly decrease (Fig. 1 bottom row).

In this paper we test the vulnerability of six real-world complex weighted networks with a total of eleven different link removal strategies. The link removal strategies are planned to consider both binary-topological and weighted network properties. For example, the betweenness centrality link removal is based on topological properties of the network, by removing links according to the higher number of shortest routes in the network passing along the links. Differently, the strong link removal deleting links according to their associated value (weight) is a weighted based strategy. To test the network robustness under link removal we adopted three widely used measures of the network functioning, the largest connected cluster (*LCC*), the network efficiency (*Eff*) and the total flow (*TF*). We chose these measures to describe both the topological (binary) and weighted structure of the network and we can see each measure like a different and not exhaustive interpretation of the network functioning. The largest connected component (*LCC*), representing the maximum number of nodes connected among them, is the simplest and widely applied indicator of the network functioning, adopted to evaluate the connectedness of Internet routers^1^, the vulnerability of power grids^10^ or as a measure of the epidemic spreading in finding the best vaccination strategies^12,31,32^. The network efficiency (*Eff*) is a widely used measure of the network functioning that can be viewed as a quantification of information spreading across the whole network where information is concurrently exchanged^2,30,33–35^. Differently from the *LCC*, the network efficiency is an indicator considering the weighted structure of the network. The total flow (*TF*) is the sum of link weights and it represents the simple measure to quantify the networks functioning considering their weighted structure^30^. In Fig. 1 we delineate the rationale behind each functioning measure by depicting simple example networks subjected to the same link removal and the associated functioning measure values.

## Methods

### The link removal strategies

- *Rand*: links are randomly removed. This represents the possibility of links failure (error) in the network^3,28,30^.
- *Strong*: links are removed in decreasing order of weight, i.e. links with higher weight are removed first^3,28,30^ and it represents an attack directed to strong links.
- *Weak*: links are deleted in increasing order of weight, i.e. links with lower weight are removed first^3,28,30^.
- *BC*: links are removed according to their betweenness centrality (*BC*), i.e. links with higher betweenness centrality are deleted first. The betweenness centrality is based on the shortest paths (also called geodesic path) between a couple of nodes. The shortest path between two nodes is the minimum number of links to travel from a node to the other^36^. The betweenness centrality of a link accounts the number of shortest paths from any couple of nodes passing along that link^36^. This version of betweenness centrality is based on the binary shortest path notion, accounting the number of links necessary to travel among nodes only, without any consideration of the weight attached to the links; for this reasons is also called binary betweenness centrality^34^.
- *BCw*: links are removed according to their weighted betweenness centrality (*BCw*), i.e. links with higher *BCw* are deleted first. The weighted betweenness centrality is computed using the weighted shortest paths that consider the number of links necessary to travel between nodes, but also consider the weight attached to the links. In this procedure, we first compute the inverse of the link weights, then we compute the weighted shortest paths as the minimum sum of the link weights necessary to travel among nodes^34,35^. The weighted betweenness centrality of a link accounts the number of weighted shortest paths from any couple of nodes (also called weighted geodesic) passing along that links^36^. The higher is the *BCw* of a link, the higher is the number of weighted shortest paths passing along the link.
- *DP*: links are removed according the degree product (*DP*) of the joined nodes. The degree of the nodes is the number of links to the nodes^5,34^. Usually the high degree nodes are the so-called hubs^1,5,34^. The *DP* pruning strategy can be viewed as a strategy ranking the links reaching information from the topological connectivity of the nodes.
- *BP*: links are deleted according to the betweenness centrality product (*BP*) of the end nodes. The betweenness centrality of a node is the number of shortest paths from any couple of nodes passing from that node^34,36^. The higher is the betweenness centrality of the node, the higher the number of shortest paths passing along the node.
- *BPw*: links are removed according the weighted betweenness centrality product (*BPw*) of the joined nodes. The weighted betweenness centrality of a node is the number of weighted shortest paths from any couple of nodes passing from that node^34,36^. The higher the weighted betweenness centrality of the node, the higher the number of weighted shortest paths passing along the node. The *BPw* is the weighted counterpart of the *BP* pruning.
- *SP*: links are deleted according to the strength product of the ending nodes. The strength of a node is the sum of the weights of the links to that node^30,34^. *SP* can be viewed as the weighted counterpart of *DP*.
- *TP*: links are deleted according to the transitivity product of the ending nodes. The node transitivity is a notion measuring the probability that the adjacent nodes of a node are connected among them. The adjacent nodes of a node are also called the ‘neighbors’ of that node. The transitivity of a node is the proportion of links between the neighbors of a node divided by the number of links that could possibly exist between them. Equally, we can compute the transitivity considering the ‘triangles’ in the network, i.e. a triangle is a subgraph of three nodes. The transitivity of a node is computed as the ratio of the closed triangles (complete subgraphs of three nodes) connected to the node and all the possible triangles centered on the node. The node transitivity is also called ‘local transitivity’ or ‘node clustering coefficient’^34,37^. See Supplemental material S1 for a detailed description. In network theory, the node transitivity is a measure of the magnitude to which nodes in a network tend to cluster together. The node transitivity defined here is a topological metric of nodes clustering not including the link weights.
- *TPw*: links are deleted according to the weighted transitivity product of the ending nodes. We adopted the weighted version of the topological node transitivity proposed by Barrat *et al*.^37^ This is also called weighted clustering coefficient of the node and it is a measure of the local cohesiveness that takes into account the importance of the clustered structure on the basis of the amount of interaction intensity found on the local triangles. Indeed, the weighted node transitivity counts for each triangle formed in the neighborhood of the node *i*, the weight of the two participating links of the node *i*. Such a measure, evaluates not only the number of closed triangles among the node *i* neighbors (like in the local binary transitivity above), but also the total relative weight of these triangles with respect to the strength of the node. See Supplemental material S1 for a detailed description. *TPw* is thus the weighted version of the transitivity product of the node (*TP)*.

In the case of ties, e.g. links with equal ranking, we randomly sort their sequence. We perform 10^3^ simulations for each link attack strategy.

We remark that the link removal strategies we used were conceived for non-directed networks, that is networks with symmetric adjacency-weight matrices. Nonetheless, all the strategies can be easily adapted for directed networks, except the *Rand*, *Weak* and *Strong* link removals. For example, the *DP* strategy that removes link according to the degree product of the ending nodes can be applied to directed network with two strategies, one ranking link according to the nodes in-degree product and the second according to the nodes out-degree product. Analogously, the *SP* strategy that removes link according to the strength product of the ending nodes can be translated to directed networks using two strategies, one ranking links according to the nodes in-strength product and the second according to the nodes out-strength product. Further, all the strategies based on the betweeness centrality can be easily adapted to their directed versions; in this case the shortest paths passing along nodes-links are directed and the travel between nodes considers the directionality of the links. Last, we can perform the directed counterparts of the nodes transitivity-based strategies adopted here by using the ‘directed nodes transitivity measure’, also known as clustering coefficient in directed networks^34^. Differently, *Weak* and *Strong* strategies that rank the links in increasing and decreasing order of weight have not a ‘directed counterpart’, since the links cannot be classified as ingoing or outgoing a node (e.g. a link outgoing a node is clearly ingoing to another). Last, the directed counterpart of the *Rand* strategy is meaningless, since the link order is a simple random sorting.

### The real-world complex networks data set

We test the efficiency of the link removal strategies using six well know real-world complex weighted networks.

(i) *US Airports flights transportation network (Air)*: This is a weighted transportation network obtained by considering the 500 US airports^38^. Nodes represent US airports and links represent air travel connections among them. The network reports the link weight expressed in terms of the number of available seats on a given connection on a yearly basis.
(ii) *The neural network of the nematode C. Elegans (Eleg)*: This biological network is a weighted representation of the neural network of *C. Elegans*^39^. Nodes are neurons and links are neural connections among them. The link weight is the number of connections between couples of neurons.
(iii) *Scientific collaboration network (Net)*: This is a social network representing the co-authorship in science publications^40^. Nodes are scholars and links depict the co-authorship relationship among them. The link weight indicates the number of co-authored papers by a couple of authors.
(iv) *Cargo ships transportation (Cargo)*: The international transportation network of global cargo ship movements consists of shipping journeys between pairs of major commercial ports in the world in 2007^41^. The link weight represents the number of shipping journeys between couples of nodes-ports.
(v) *The Escherichia Coli metabolic network* (*Coli)*: this biological network illustrates the common chemical reactions between metabolites in the *E. Coli* bacteria. Nodes are metabolites and links indicate the presence of common reactions. Link weights in the metabolic network of the bacteria *E. Coli* consist of the number of different metabolic reactions, in which two metabolites participate^42^.
(vi) *The UK faculty social network (UK)*: This social network represents the friendship among academic staff in a UK faculty. The personal friendship network of the UK faculty university consists of 81 nodes (individuals) and 817 weighted friendship connections^43^. The network structure was constructed with a questionnaire, where the staff individuals formed a reliable scale and declared the strength of the friendship with other individuals in the faculty. The links weights are thus representing the strength of the friendship among individuals.

First, we selected this database because it is composed by the real-world weighted networks well known in literature and they are used in yet classic analyses. Second, they describe different realms from different fields of science with a widely different but solid interpretation of link weight. Last, the networks are of different structural properties, such as size (e.g. number of nodes, from *N* = 81 to *N* = 1589), number of links (from *L* = 817 to *L* = 4349) and connectivity level (average node degree <*k* > from 3.45 to 20.2). The real-world networks data set description and main structural features are in Table 1.

**Table 1:** Real-world complex networks features.

Name	US Airports	*C. Elegans*	Netscience	Cargo ship	*E. Coli*	UK faculty
Type	Transportation	Biological	Social	Transportation	Biological	Social
Nodes	Airports	Neurons	Scientists	Ports	Metabolites	Individuals
Links	Airport Routes	Neurons Connections	Co-autorship	Port Routes	Common reactions	Friendship
Weights	Passengers	Connections number	Common papers	Shipping journeys	Common reactions	Friendship Strength
*N*	500	279	1589	834	1100	81
*L*	2980	2287	2743	4349	3637	817
<*k*>	11.9	15.3	3.45	10.4	6.6	20.2
*k*_*min*_, *k*_*max*_	1,145	0,134	0,34	0,173	1,152	2,62
<*w*>	1815657	57.6	1.5	897.44	9.01	92.1
*w*_*min*_, *w*_*max*_	9416,49316361	0,1700	0, 30	0,24931	1,229	2,379
<*E*_*w*_>	152320.2	3.76	0.43	97.70	1.36	4.57
<*l*>	2.99	2.46	5.82	3.34	3.84	2.1
Ref.	37	38	39	40	41	42
Acronym	*Air*	*Eleg*	*Net*	*Cargo*	*Coli*	*UK*

*N* number of nodes; *L* number of links; <*k*> average node degree; *k*_*min*_ minimum node degree, *k*_*max*_ maximum node degree; <*w*> average node strength; *w*_*min*_: minimum node strength, *w*_*max*_ maximum node strength, <*E*_*w*_ > average links weight, *l* average paths length.

### The network functioning measures

#### The largest connected cluster (*LCC*)

The largest connected cluster (*LCC*) is a widely used measure of the network functioning^1,4–6^. The *LCC* is also known as the giant component (or giant cluster) and it is the highest number of connected nodes in the network. The *LCC* can be written:

1 $$LCC=\,\max ({S}_{j})$$

where *S*_*j*_ is the size (number of nodes) of the *j*-th cluster.

Although the wide range of application, the *LCC* owns important shortcomings, for example by neglecting the other lower size nodes clusters and more important, neglecting the heterogeneity in the link weights^30,35,44^. The *LCC* is a simple indicator evaluating the binary-topological connectedness of the network; for this reason we adopt it like a measure of the simple topological connectivity of the network functioning not reflecting the heterogeneity of the link weights.

#### The total flow (*TF*)

The total flow represents the actual or the potential flowing in the network^30^ and it is the sum of link weights. Let be the weighted network G_w,_ it can be represented by a *N × N* matrix W where the element *w*_*ij*_ > 0 if there is a link of weight *w* between nodes *i* and *j*,and *w*_*ij*_ = 0 otherwise.

The total flow is:

2 $$TF=\mathop{\sum }\limits_{i=1}^{N}\,\mathop{\sum }\limits_{j=1}^{N}{w}_{i,j}$$

For example, in the US Airports the *TF* measure represents the actual flows among airports (where ‘actual’ means the flying passengers in a year); also in the transportation Cargo ship network *TF* represent the actual flow indicating the shipping journeys between ports in a year. Differently, in the *C. Elegans* real-world complex weighted network, *TF* indicates the total number of connections realized between pairs of neurons. In other terms, *TF* can be viewed as the thermodynamics capacity or a quantity influencing the actual flow between nodes pairs in the network but do not uniquely determine it, e.g. the higher is the connection density in the *C. Elegans* network, the higher can be the information delivered between couple of neurons. The *TF* is the simplest weighted indicator of the network functioning, only quantifying the weight value of the removed links, neglecting their topological role in the network.

#### The efficiency (*Eff*)

The concept of efficiency of the network was first introduced by Latora and Marchiori^2^ with the aim to encompass specific shortcomings associated to the shortest path based measures. In fact, the shortest path based measures, like the characteristic path length or the average geodesic length^2,34^, can be divergent when the network is not connected. For this reason, these measures based on the paths presents the shortcoming to diverge for disconnected networks making them poorly suited to evaluate network functioning under nodes-links removal. Differently, the network efficiency (*Eff*) can properly evaluate the functioning of both connected and disconnected networks, and this becomes a highly important property when we have to measure the network functioning under nodes-links attack. After this, the network efficiency can properly work with both binary and weighted structures, being able to consider the difference in link weights in the evaluation of the weighted network functioning. The efficiency of a network is a measure of how efficiently it exchanges information. On a global scale, i.e. considering all the nodes-components of the system, the efficiency quantifies the exchange of information across the whole network where information is concurrently exchanged. The efficiency is a robust and widely used weighted measure of the network functioning adopted in very different fields of science^2,30,33–35^. The average efficiency of the network is defined:

3 $$Eff=\frac{1}{N\cdot (N-1)}\,\sum _{i\ne j\in G}\frac{1}{d(i,j)}$$

where *N* is the total number of nodes and *d(i,j)* is the shortest path between node *i* and node *j*. In our analyses we adopted the weighted version of the efficiency metric with *d(i,j)* representing the weighted shortest path between node *i* and node *j*. To calculate the weighted shortest paths, we first applied a standard procedure by computing the inverse of the link weights^30,34,35^. This standard procedure has the aim to consider ‘shorter and wider routes’ the links of higher weight and ‘longer and narrow routes’ the links of lower weight. As a consequence, the procedure evaluates as ‘tightly connected’ or ‘less distant’ the couples of nodes joined by the higher link weights. The weighted shortest path between two nodes will become the smallest sum of the inverse links weight necessary to travel between the nodes (with the links of higher weight representing ‘faster and of high delivery efficiency’ routes). This procedure is intended to consider in real-world networks strong links as more important for the network functioning with the weight of the link acting as an indicator of transport capacity-efficiency between the connected nodes. For example, in the US Airports the link weights represent the passenger flowing among airports in a year and, in this system, higher link weights indicate routes among pairs of airports with higher transportation capacity in terms of passengers. In the transportation Cargo ship network, the link weight accounts the shipping journeys flowing between ports in a year and the it can be viewed as an indicator of the mass transport capacity between two ports. Analogously, in the *C. Elegans* real-world complex weighted network, the link weight counts the total number of connections realized between pairs of neurons and it can be viewed as a quantity influencing the information signal flowing between neurons, e.g. the higher the connection density in the *C. Elegans* network, the higher can be the information delivered between couple of neurons. Once the weighted shortest paths are computed, the weighted network efficiency is the sum of the inverse of the weighted shortest paths among couples of nodes, with shorter paths producing higher functioning efficiency (*Eff*) in the network. For a detailed explanation of the weighted shortest path notion and of the related weighted efficiency measurement see Bellingeri *et al*.^30^

### Ranking the efficacy of the link removal strategies

**Figure 2:**
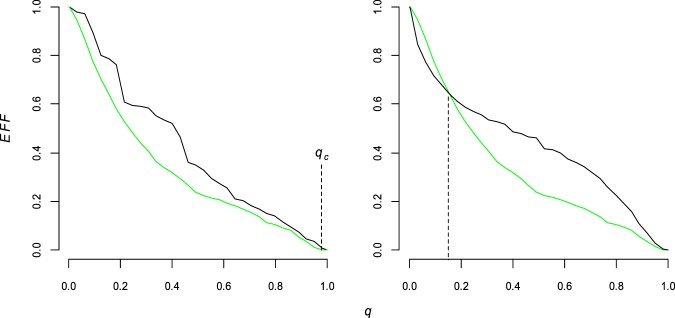
The network functioning along the link removal process. Functioning efficiency measure (*Eff*) as a function of the fraction of link removed (*q*) for different attack strategies. The examples give in this chart are from the UK faculty network. Left chart: *Strong* strategy (green line) triggers a faster efficiency (*Eff*) decrease than the *DP* strategy (black line) and the robustness area (*R*) below the green curve is lower than the one below the black curve. The widely used percolation threshold *q*_*c*_ is roughly the same for the two strategies (*q* = 0.98, vertical dashed) and this measure of the network functioning is not able to individuate the difference. Right chart: in this simulation for *q* = 0.16 (abscissa of the vertical dashed line) we observe a cross between *Strong* (green) and the *BC* (black) strategy curves; this means that the black strategy is more harmful at the beginning of the removal process (before *q* = 0.16) and the green strategy is more efficacy after *q* = 0.16. The robustness area resuming the entire process in a single value is not able to evaluate the local efficacy of the strategy; to understand the efficacy of the attack strategies in the first fraction of the removal process we add a comparison for three small values of *q* = (0.05, 0.1, 0.15).

We consider the best link removal strategy as the one able to produce the faster functioning decrease in the network. In other words, the strategy able to select most important links in the networks. To evaluate the decrease in the network functioning we follow two ways. First, we consider the global functioning decrease along the removal process by computing the area below the curve of the measure of network functioning subjected to link removal. This is the analogous to what has been done in Schneider *et al*.^45^ where the authors used the largest connected component (*LCC*) parameter to evaluate the network functioning damage triggered by an intentional attack directed to the nodes. This procedure has the merit to resume the damage in a single number that Schneider *et al*.^45^ called robustness of the network (*R*). Faster decrease in the network functioning measure (for example the *LCC* in Schneider *et al*.^44^) returns lower *R* values indicating higher damage caused in the networks. The best attack strategies are those producing lowest *R* and thus the ones selecting most important components in the networks. We applied the robustness *R* as a global measure to evaluate the decrease of the three indicators of the networks functioning *Eff*, *LCC* and *TF* along the removal process. Nonetheless, it has been shown that the damage produced by the nodes attack strategies depends on the number of nodes removed in the network^30,31,46^. This means that comparing two strategies, e.g. A and B strategies, A can be more harmful than B when removing 10% of the nodes, yet strategy B becomes more efficient than A to decrease the network functioning when removing the 40% of the nodes^31,46^. The *R* measure is not fully able to compare the efficacy of the compared strategies in this case. For this reason, we also evaluate the link removal strategy in the first stages of the removal process, computing the decrease in the network functioning measures for 5%, 10% and 15% of links removal. To evaluate the removal process for narrow fraction of removals is particularly important because partial malfunctioning affecting a small amount links-components are more probable than the global destruction of the network represented by removing all the links. Adopting the two ways for quantifying the decrease in the network functioning measurements we present a thorough evaluation of how the link removal strategies are efficient along the whole removal process. One of the oldest indicator of network robustness under nodes-links removal is the percolation threshold *q*_*c*_ indicating the removals fraction of nodes or links necessary to completely vanish the *LCC*^1^. However, the percolation threshold *q*_*c*_ is inaccurate to fully describe the decrease in the network functioning owing the shortcoming to completely neglect the vulnerability of the network along the removal process^30,31,46^. In Fig. 2 we give an example of link removal and the associated robustness measure (*R*).

## Results and Discussion

### The network robustness against the link attack strategies

#### Eff

**Figure 3:**
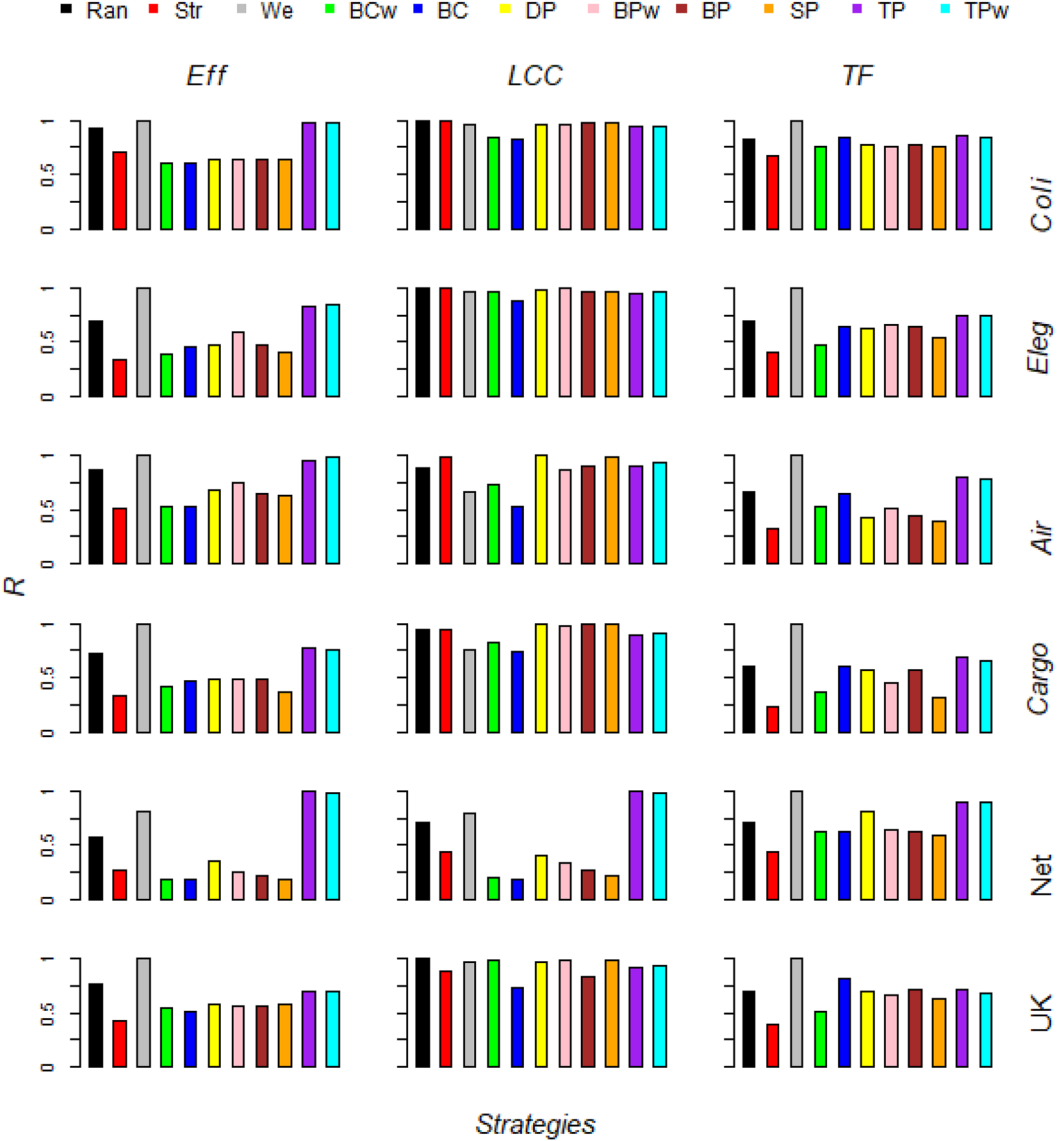
Real-world complex networks robustness *vs* link removal strategies. The robustness *R* of the functioning measurements *Eff, LCC* and *TF* along the whole link removal process for each link attack strategy for the six real-world networks. The network robustness is normalized by the max robustness for that system functioning measure. The lower is *R*, the higher is the efficacy of that link attack strategy to damage the network. Link removal strategies: random (*Ran*), strong (*Str*), weak (*We*), link weighted betwenness centrality (*BCw*), link binary betwenness centrality (*BC*), end nodes end nodes degree product (*DP*), end nodes betwenness centrality product (*BPw*), end nodes betwenness centrality product (*BPw*), end nodes strength product (*SP*), end nodes binary transitivity product (*TP*), end nodes weighted transitivity product (*TPw*).

**Figure 4:**
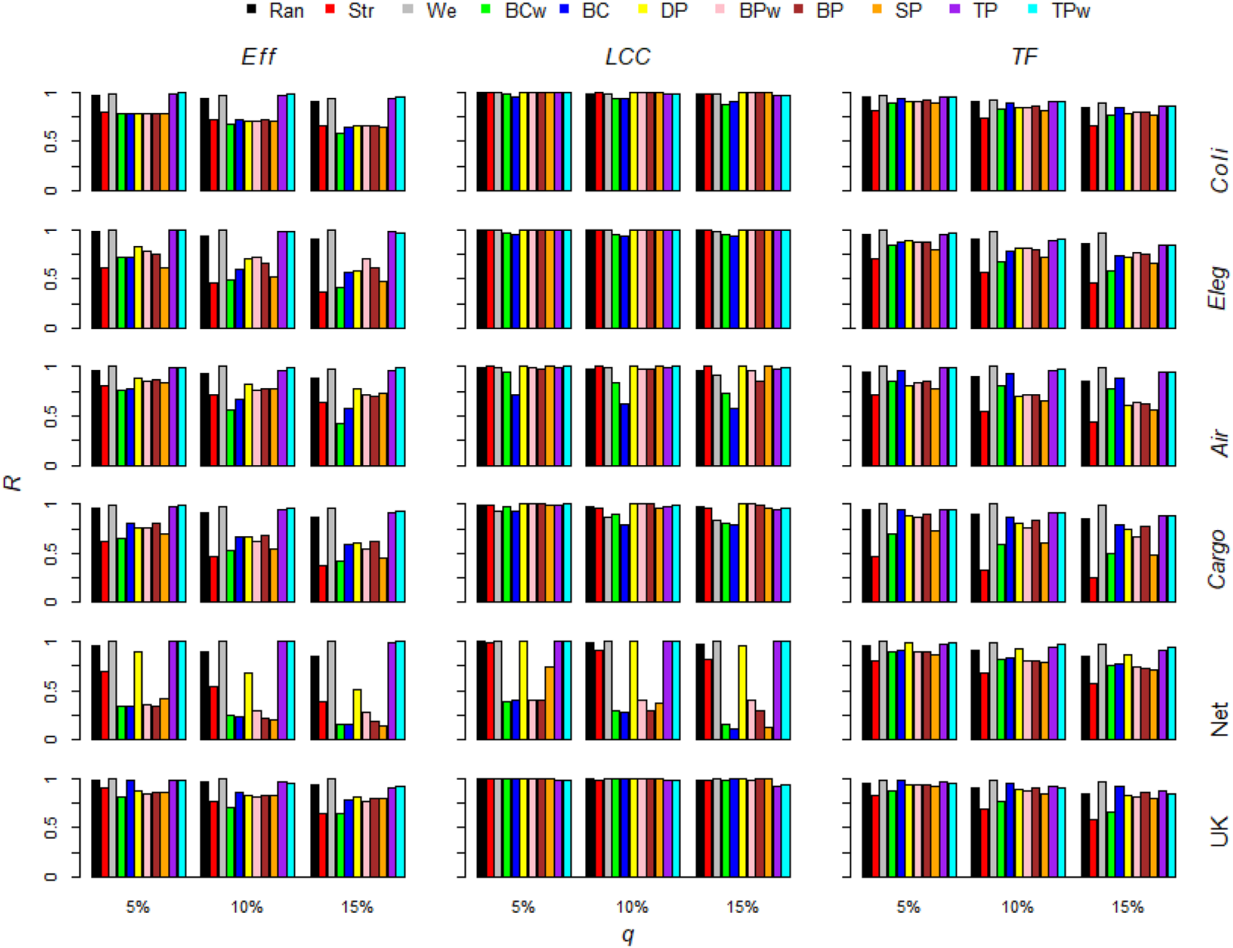
Real-world complex networks robustness *vs* link removal strategies after small fraction of links removed. The robustness *R* of the functioning measurements *Eff, LCC* and *TF* after *q* = 5, 10, and 15% removed links for each links attack strategy for each real-world networks analyzed. The network robustness is normalized by the max robustness for that system functioning measure. The lower is *R*, the higher is the efficacy of that link attack strategy to damage the network. Link removal strategies: random (*Ran*), strong (*Str*), weak (*We*), link weighted betwenness centrality (*BCw*), link binary betwenness centrality (*BC*), end nodes end nodes degree product (*DP*), end nodes betwenness centrality product (*BPw*), end nodes betwenness centrality product (*BPw*), end nodes strength product (*SP*), end nodes binary transitivity product (*TP*), end nodes weighted transitivity product (*TPw*).

The link removal strategies based on the weight of the links (*Strong*) and on the betweenness centrality (*BCw* and *BC*) are the best to decrease *Eff*. When the robustness is computed along the entire removal process the *BCw* and *BC* strategies are the most effective in 2 out 6 of cases. *Strong* strategy is the best in the others 4 out 6 (Fig. 3 and Table 2). Even when the robustness is computed at the beginning of the removal process (5%, 10% and 15% of links removal), we generally found *Strong* and *BCw* more efficient than the other strategies (Fig. 4 and Table 3). The network efficiency (*Eff*) evaluates the information spreading in the system and it is shaped by two main factors, the topological (binary) and the weighted structure of the network. The topological structure is of high efficiency when links are distributed among nodes forming short paths in the networks. Many real-world networks have been found to own an efficient topological structure^2,46^ and many analyses focused the network features increasing the information spreading, such as the small-world phenomenon^13,34^. Differently, the weighted structure of the network can shape higher information spreading by presenting higher link weights (e.g. shortening the nodes pairs distance) and by delivering these strong links along the topological shortest paths (e.g. shortening the average distance among each nodes pairs). The finding that the weighted link removal strategies such as *BCw* and *Strong* are the best to decrease *Eff* would indicate that the weighted structure of the networks may play an important role into support the information delivery efficiency in real-world systems. The best link removal strategies following *BCw* and *Strong* are the *SP* and the *BPw*. Taken together these findings indicate that, while the aim is to decrease the efficiency (*Eff*) of the real-world complex networks, the best methods to remove link are based on the link weight and on the link betweenness centrality.

**Table 2:** The three best strategy to decrease the real-world networks functioning measurements (i.e. *Eff*, *LCC* and *TF*) measured by the robustness area for each real-world networks.

*E. Coli*	*C. Elegans*	US Airports	Cargoship	Netscience	UK Faculty
***Eff***					
*BCw*	*Strong*	*Strong*	*Strong*	*BC*	*Strong*
*BC*	*BCw*	*BCw*	*SP*	*SP*	*BC*
*BP*	*SP*	*BC*	*BCw*	*BCw*	*BCw*
***LCC***					
*BC*	*BC*	*BC*	*BC*	*BC*	*BC*
*BCw*	*TP*	*Weak*	*Weak*	*BCw*	*BP*
*TP*	*SP*	*BCw*	*BCw*	*SP*	*Strong*
***TF***					
*SP*	*BCw*	*SP*	*SP*	*SP*	*BCw*
*BCw*	*SP*	*DP*	*BCw*	*BCw*	*SP*
*BPw*	*DP*	*BP*	*BPw*	*BC*	*BPw*

**Table 3:** Best strategy to decrease the real-world networks functioning measurements (i.e. *Eff*, *LCC* and *TF*) for 5, 10, 15% of links removal.

% Removals	*E. Coli*	*C. Elegans*	US Airports	Cargoship	Netscience	UK Faculty
***Eff***						
5%	*SP*	*Strong*	*BCw*	*Strong*	*BP*	*BCw*
10%	*BCw*	*Strong*	*BCw*	*Strong*	*SP*	*BCw*
15%	*BCw*	*Strong*	*BCw*	*Strong*	*SP*	*BCw*
***LCC***						
5%	*BC*	*BC*	*BC*	*Weak*	*BCw*	*TP*
10%	*BC*	*BC*	*BC*	*BC*	*BC*	*TP*
15%	*BCw*	*BC*	*BC*	*BC*	*BC*	*TP*
***TF***						
5%	*BCw*	*SP*	*SP*	*BCw*	*SP*	*BCw*
10%	*SP*	*BCw*	*SP*	*BCw*	*SP*	*BCw*
15%	*SP*	*BCw*	*SP*	*SP*	*SP*	*BCw*

#### LCC

In all the six real-world complex networks we analyzed here, the *BC* strategy is the most efficient to vanish the *LCC* (Fig. 3 and Table 2). This finding confirms, on the side of link removal strategies, recent outcomes of a large benchmark comparison of the widely used nodes attack strategies showing how the recalculated nodes betweenness centrality attack is the best attack in 80% of the case, both in real and model networks^6^. Our and Wandelt *et al*.^6^ outcomes indicate that the betweenness centrality removal of the nodes and links is highly efficient because the definition of the betweenness is extremely well aligned with the aim to disrupt the main communication paths of the network thus triggering the faster fragmentation of the *LCC*. Nonetheless, the link removal strategy based on the nodes betweenness centrality, e.g. the *BP* that removes links according to the betweenness product of the ends nodes, is clearly less efficient than the *BC* link removal strategy, indicating that to individuate most central links raising information from the betweenness centrality of the ends nodes, may degrade the betweenness centrality properties of the ranked links, then resulting in a worsen efficacy into fragment the *LCC* (Fig. 3). This last outcome would indicate that to select most important links sustaining the global topological connectivity of the networks is fundamental to sample direct information properties from the links; in the case this is not possible, and only nodes properties are available, the resulting important links ranking would be less reliable. We outline that our *BC* removal strategy is computed on the initial networks (e.g. before any link deletion). Many analyses showed that after nodes removal the betweenness properties of the remaining network components (both nodes and links) may change and thus the recalculated (adaptive) betweenness nodes attack is more efficacy than the non-recalculated counterpart^5,6,46^. For this reason, it will be a straightforward extension of the analyses presented in this paper to implement recalculated (adaptive) removal strategies based on the betweenness centrality that can be able to individuate changes in the network structure.

In all the six real-world networks we analyzed here, to add information on the link weights by deleting links according to the weighted betweenness centrality (*BCw*) worsen the efficacy into fragment the *LCC* with respect the binary link removal strategy *BC* (Fig. 3). For example, in the *UK* network *BC* removal strategy is the best method to fragment the *LCC* where instead *BCw* performs similar to the random removal of links *Rand* (Fig. 3). The higher *BC* link removal strategy efficacy to reduce the *LCC* is found even at the starting of the removal process, even less significant for *Coli*, *Eleg* and *UK* networks (Fig. 4). The higher *BC* efficacy we found in many real-world complex networks indicated that with the aim to reduce the network *LCC*, including link weights information can reduce the effectiveness of the removal strategies into select important links for the topological connectedness of the network. Many applications of network science from protection of power grid networks^10^ to vaccination plans halting epidemic spreading^12,31^ are considered mathematically equivalent to find the fastest *LCC* fragmentation; our findings indicate that with the aim to reduce the *LCC*, considering the link weights would be not useful and it would even worsen the selection of the most important links to the network connectedness, i.e. the links with higher betweenness centrality.

The role of the weak links in sustaining the cohesiveness of the system was already emphasized in the classic sociological paper of Granovetter^23^ which showed how weak acquaintances relationship play the role to connect communities far apart in social networks. Recent network theory studies confirmed this hypothesis showing that the largest connected cluster (*LCC*) is highly vulnerable to the removal of links with lower weight (weak links) but robust to deletion of links of higher weight (strong links)^24–28^. On the contrary, the strong link removal triggers a faster (*LCC*) fragmentation in science co-authorship networks (*Net*)^30,47^. In this scientific social network, dense local nodes neighborhoods mainly consist of weak links, and the strong links depicting more intense and long-term relationships between leader scholars join far apart research communities thus resulting more important for overall network connectivity^48^. We found higher vulnerability to weak link removal only for the transportation networks, such as the *Cargo* and *Air* (Fig. 3). In the others real-world networks *Weak* strategy triggers similar *LCC* decrease than *Strong* (*Coli* and *Eleg* networks) whereas in the social networks *Net* and *UK* to delete weak links causes slower *LCC* fragmentation. Even though in all real-world complex networks we analyzed, the *BC* strategy removing links according to the binary betweenness centrality of the links produced the faster *LCC* disruption (Fig. 3). This finding indicates that the links with higher betweenness centrality, i.e. the ones driving most of the shortest routes in the network, are the true key players of the real-world network topological connectivity. For this reason, we bring an interesting remark inside the long-standing debate about weak-strong link importance, indicating that the links playing the major role into sustaining the cohesiveness of the system are clearly the ones driving most of the shortest routes in the network, not necessarily the weakest or the strongest links.

#### TF

When we focus the link removal problem with the aim to decrease the total flow (*TF*) in the networks, *Strong* strategy removing links in decreasing order of weight is the best strategy by definition (Figs. 3 and 4). In fact, the best solution of sorting links producing the faster total flow (total weights) decrease is mathematically equivalent to order a numerical vector in decreasing order of values. For this reason in Table 2 we rank the efficacy of the link removal strategies keeping out the *Strong* strategy; we then adopt the *Strong* outcomes as a benchmark comparison for the other strategies. For the whole removal process, in 2 out of 6 cases, the best methodology is the *BCw* strategy. This finding means that the links with higher weighted betweenness centrality, e.g. the more central links where passes the higher number of shortest routes among nodes, are also links owing higher weight. The higher efficacy of the *BCw* strategy is found in the *Eleg* biological network and for the social network *UK* (Fig. 4, Table 2). Neuronal networks are systems for the information delivery and they are expected to evolve toward higher functioning level. For this reason, we hypothesize that the *C. Elegans* neuronal networks evolved more central links playing the major role in the information delivery with higher number of connections (e.g. higher link weight). Further, the *BCw* is clearly more efficient than other strategies in the UK faculty social network. The higher efficacy of the *BCw* into decrease the total flow indicates that in the *UK* network links with higher weight are more likely to be those more central (higher weighted betweenness centrality). Translating this outcome into social network terms, it would indicate that stronger friendship relationship between individuals are likely to be the more central in this social network; since the link centrality computed with weighted betweenness is shaped by both the topological and weighted embedding of the link in the network, with an intricate interaction of these two factors, further future investigations will be necessary to shed light on this complex relationship emerging in the structure of weighted networks.

In 4 out of 6, the best strategy is the *SP* deleting links with higher strength product of the end nodes. We find this for the two transportation networks, i.e. *Air* and *Cargo* (Figs. 3 and 4). Given that the strength of the node is the sum of the link weights to it^34,35^, the finding that in real-world transportation networks the links connecting nodes with higher strength are even more likely to be of higher weight indicates that the connection routes between the bigger airports or ports are also the wider in terms of passengers or boat shipping. Then, we find *SP* the most efficient strategy to decrease *TF* in the *Coli* real-world network representing the metabolites system of the *E. Coli* bacteria, e.g. the nodes are metabolites and links depict common reactions among them. The higher strength nodes are the metabolites involving the highest number of reactions in the *Coli* metabolic network and they can be viewed as the most common metabolites. Thus, to have higher *SP* links with higher weight would indicate that the connections between most common metabolites are also the links indicating higher activity level (higher number of common reactions) between those metabolites. However, the *SP* is only slightly more efficient than the following removal strategies (Figs. 3 and 4). Even for the *Net* network, the best strategy is the *SP* that removes links according to the strength product of the end nodes. This finding depicts a specific structure for the science co-authorship network (*Net*) for which the strong links, that represent the scientific collaborations with higher number of common papers, are positioned among the most prolific scholars, e.g. the nodes of higher strength.

#### Comparing the measures of network functioning

**Figure 5:**
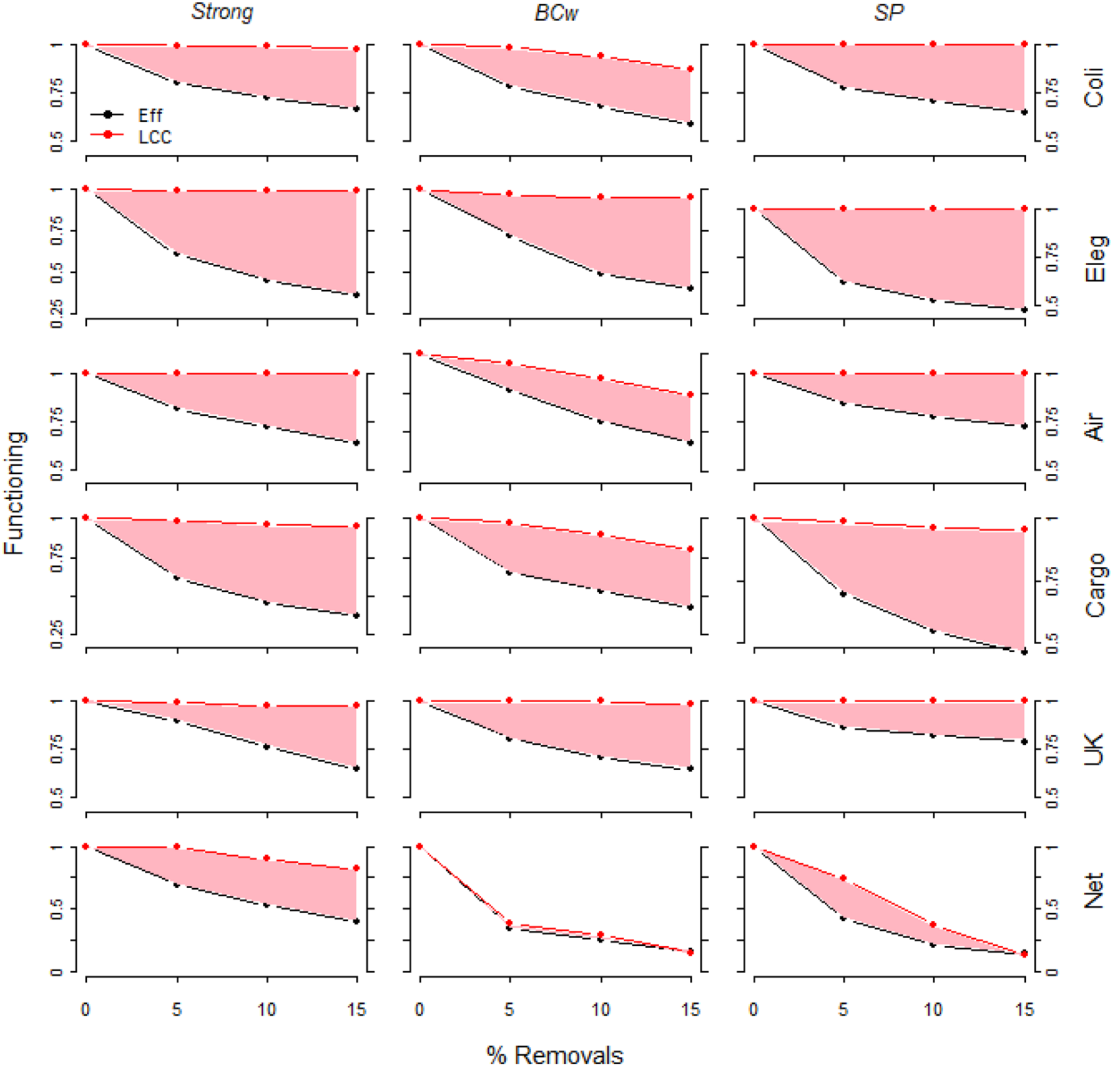
Real-world complex weighted networks functioning decrease (*Eff* & *LCC*) under 5, 10, 15% of links removed. The system functioning is depicted under link removal for the three most harmful link attack strategies, e.g. *Strong*, *BCw* and *SP*. The system functioning is normalized by the initial functioning value (e.g. before any removal). The pink area depicts the difference between *Eff* and *LCC* measures along the link removal process. For all networks except *Net*, under *BCw* and *SP* link removal strategies, after small fraction of links removed we observe a quick efficiency (*Eff*) decrease whereas the largest connected cluster (*LCC*) decreases very slowly.

**Figure 6:**
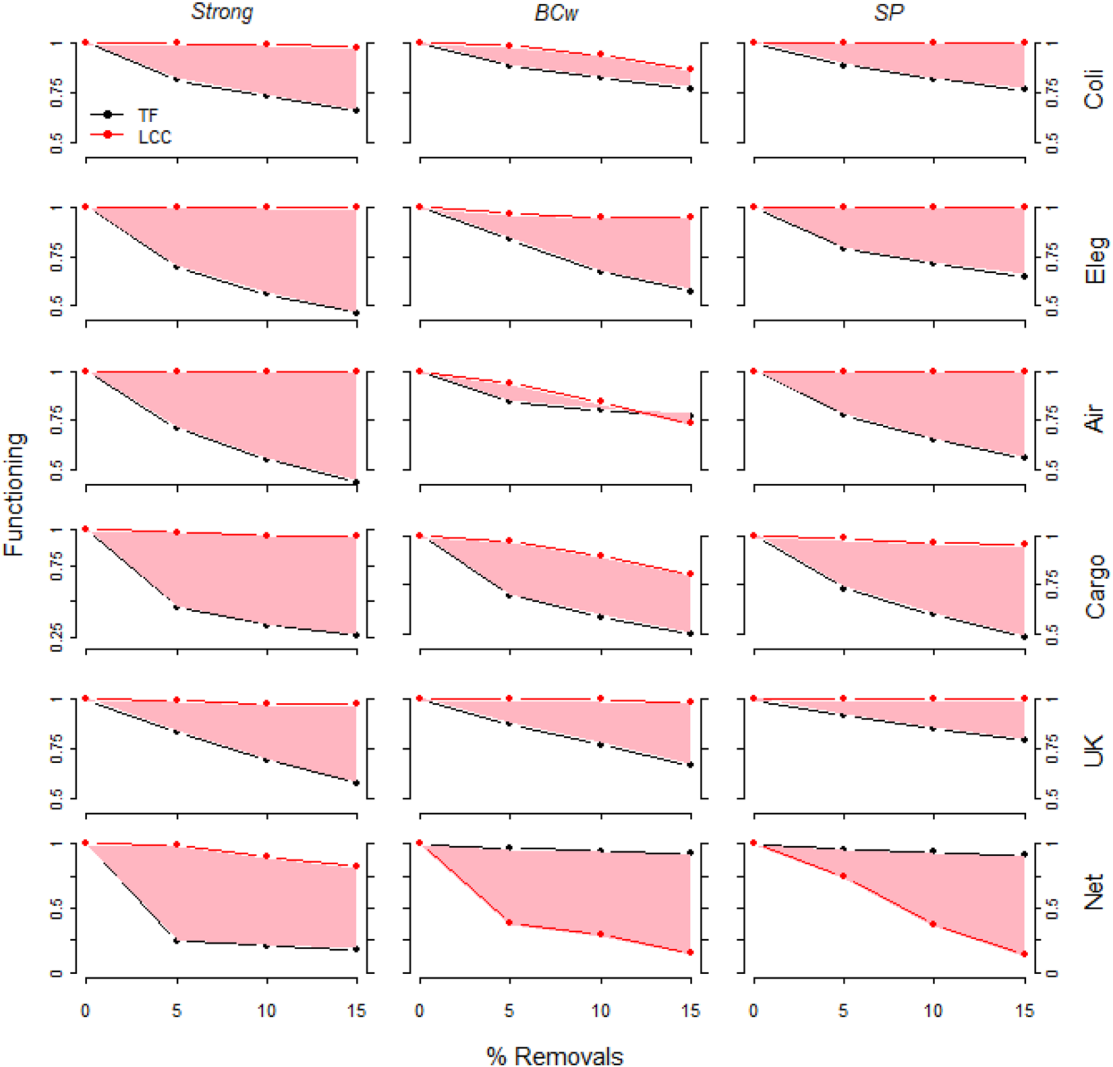
Real-world complex weighted networks functioning decrease (*TF* & *LCC*) under q = 5, 10, 15% of links removed. The system functioning is depicted under link removal for the three most harmful link attack strategies, e.g. *Strong*, *BCw* and *SP*. The system functioning is normalized by the initial functioning value (e.g. before any removal). The pink area depicts the difference between *TF* and *LCC* measures along the link removal process. For all networks except *Net*, under *BCw* and *SP* link removal strategies, after small fraction of links removed we observe a quick efficiency (*TF*) decrease whereas the largest connected cluster (*LCC*) decreases very slowly. In the Netscience network under *BCw* and *SP* link removal we find the opposite pattern: *TF* remains roughly constant and the *LCC* sharply decreases.

**Figure 7:**
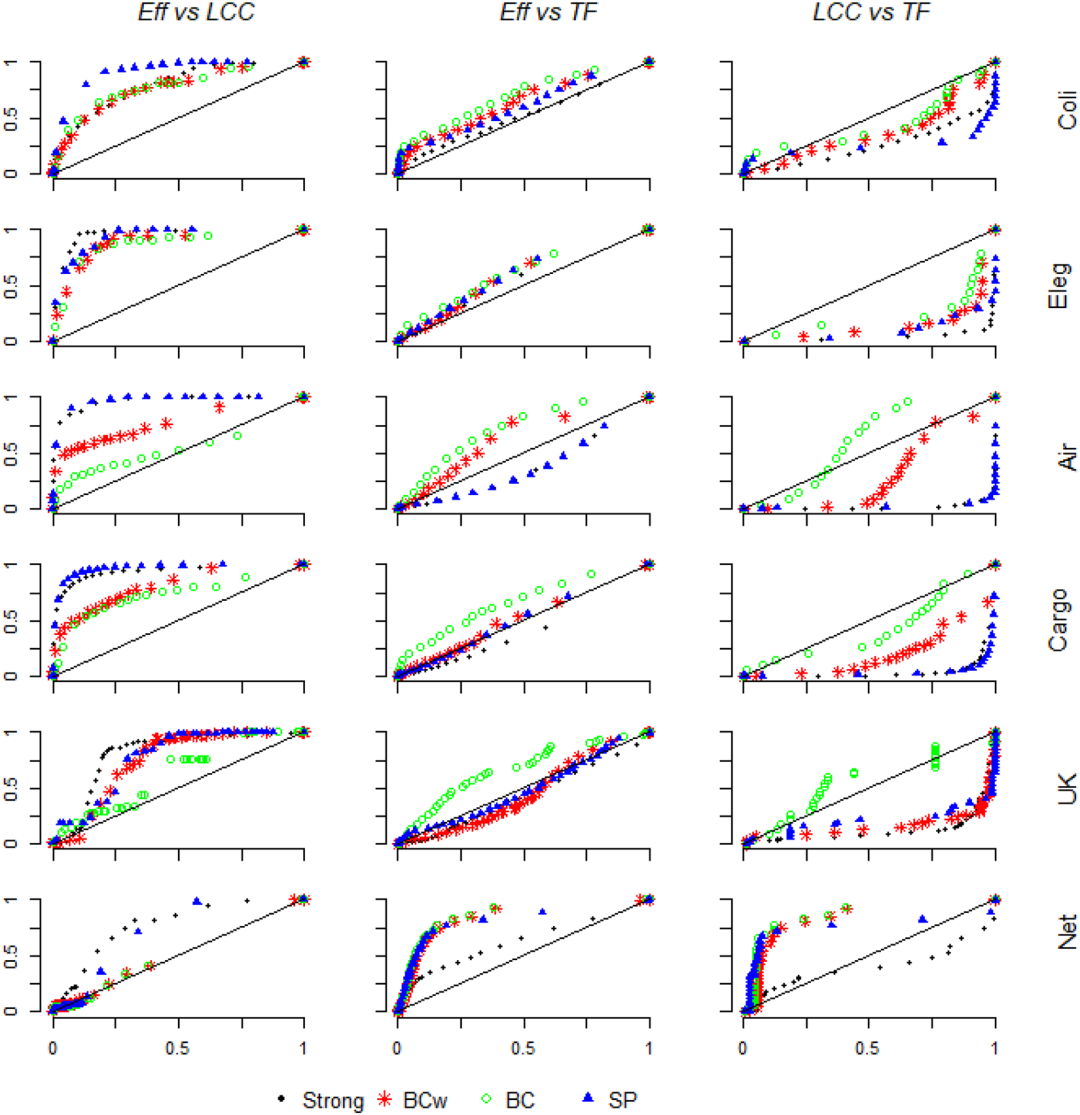
Real-world complex weighted networks functioning comparison. The measures of system functioning are plotted along the whole link removal for four harmful link attack strategies, e.g. *Strong*, *BC*, *BCw* and *SP*. The system functioning is normalized by the initial functioning value (e.g. before any removal). The bisector line indicates the perfect correlation between the two measures, e.g. the network response turned out by the measures is the same. The more the measures comparison is distant from the bisector line, the higher is the discrepancy of the system response furnished by the measures. For example, in the *Eff* vs *LCC* we see most of the comparison lying above the bisector line, indicating the faster decrease *Eff* decrease under the link removal strategies.

For most of the strategies and most of the real-world networks, we find an important difference between the network functioning measures *LCC* and *Eff* when removing 5, 10, 15% of links (Figs. 5 and S1 in Supplemental material). This difference is bigger for the removal strategies selecting highest link weights (*Strong)* and for the strategies removing link connecting higher strength (*SP*) and weighted betweenness nodes (*BPw*). For example, in *Cargo* and *Eleg* following the removal of 15% of links we observe *Eff* collapsing below the 50% of the initial value where instead the *LCC* measure does not decrease (Fig. 5, *Strong* column). Further in *Coli* network the removal of the 15% of highest *SP* links triggers the *Eff* decrease below the 60% of the initial value. Only in the *Net* network, the *LCC* follows the *Eff* trend, especially with *BC* strategy (Fig. 5). This would confirm that in the science co-authorship network (*Net*) the links of highest weight play a fundamental role in sustaining system connectedness. The difference between the *LCC* and *TF* measures is even bigger: e.g. when removing 15% of strong links *TF* falls to the 25% of the initial value in *Cargo* and *Net* networks (Fig. 6, *Strong* column and Fig. S2 of the Supplemental material). Recent outcomes showing how five nodes attack can trigger an abrupt collapse of the weighted functioning measures (*Eff* and *TF*) while the *LCC* parameter that evaluate the simple binary connectedness of real-world complex weighted networks are almost unaffected, i.e. the attack toward few highest degree and strength nodes returns real-world systems in a *connected but inefficient state*^30^. The findings we present in this paper confirm and aggravate the measure gap in evaluating the network functioning, showing how the removal of a small fraction of links connecting higher betweenness, higher degree or higher strength nodes, in most of cases does not affect the *LCC* size yet quickly collapsing the network efficiency *Eff* and the total flow *TF*. This evidence outlines how to adopt the simple network connectivity may be a misleading measure of the real-world networks integrity in the most likely case of real-world malfunctioning, e.g. when failure or attack occur with the system yet globally connected. Last, to furnish a complete parallel measure comparison of the network response under link removal, we depict the scatter plots of the normalized functioning measures in Fig. 7 for four harmful link attack strategies, e.g. *Strong*, *BC*, *BCw* and *SP*. The bisector line indicates the theoretical case of complete correlation between the two measures; in this ideal case the network response turned out by the different functioning indicators (*Eff*, *LCC*, and *TF*) is the same. We find strong decorrelation for the *Eff* vs *LCC* coupling, with most of the comparisons lying above the bisector line, indicating the sharper efficiency (*Eff*) decrease (Fig. 7, left column). Differently, we observe a good *Eff* vs *TF* correlation with most of the trends approaching the bisector lines. The last scatter plot depicting *LCC* vs *TF* clearly outline high level of decorrelation between the two measures of functioning with very faster decrease in the total flow of the network with associated very slow *LCC* fragmentation (Fig. 7, most of the comparisons are below the bisector line).

## Conclusions

In this paper we report the largest comparison in our knowledge of link attack strategies efficacy, by testing eleven different strategies over six real-world networks. We summarize the three main outcomes. First, the links removal strategies based on the binary betweenness centrality is the best method to fragment the *LCC;* to find the best links-nodes removal strategy to vanish the *LCC* is a central problem in complex network theory^1,4–6,20–22,46^, our outcomes show that the links removal strategy removing higher betweenness links is the best strategy to fragment the *LCC* thus indicating that the betweenness centrality is probably the most important feature to identify the nodes-links fundamental for the network connectedness. This outcome also places an interesting remark within the ‘weak-strong link importance’ classic debate, showing that the links playing the major role into sustaining the real-world networks connectivity are clearly the ones with highest betweenness, and they are not necessarily the weakest or the strongest links. Second, the removal strategy based on the weighted properties of the links, such as *BCw* and *Strong*, are the most efficient to decrease the network efficiency; since the efficiency (*Eff*) is a measure formed by the contribution of both the topological (binary) and the weighted structure of the network, this last outcome unveils that the weighted nature of the links may play a more important role into shaping the global system information spreading. Third, when removing a small strong links fraction we assist to the quick fall of the weighted measures of network functioning *Eff* and *TF* while the *LCC* indicator of the topological connectivity still holds to the initial value. Since real-world networks malfunctioning is likely to occur with the system still connected, as for example the case of routes closure in a transportation networks with locations still reachable but with longer or congested paths, our outcomes outline that to well evaluate the link importance in real-world networks it is necessary to *i*) adopt weighted measures of network functioning and *ii*) analyze the system response to reduced amount of removed links. Last, we outline that to protect nodes in real-world networks turns out to be easier than preserving the links, for instance it is easier to garrison the train stations than the railways, or it can be possible to protect the banks rather than to secure all the routes an armored car has to travel. Given the concrete difficult to protect link-connections rather than nodes in real-world networks, it turns out be even more important to focus on protecting fundamental links for the system functioning.

The analyses presented here may open future researches, such as by further investigating the role of the coupling between the topological and the weighted structure in shaping the network robustness, for example by checking the efficacy of different link removals over model networks when specific structural parameters are tuned. For example, the weighted random graphs^28^ and the Hopfield-like models for weighted neural^49^ and social^50^ networks, show non-random association between the topological and weighted structure inducing higher connectivity robustness under strong links removal. Yet, such an analysis is out of the aim of the present work, it can be very interesting to test the response of these model networks under some of the different link removals strategies proposed in this paper with the aim to shed light on the causes of the real-world weighted networks robustness.

## Supplementary information

Supplementary Information.

Supplementary Information2.
