# Supplementary material for: A comparative analysis of link removal strategies in real complex weighted networks
Source: Sci Rep. 2020 Mar 3;10:3911. doi: 10.1038/s41598-020-60298-7 (PMC7054356; doi:10.1038/s41598-020-60298-7)
Supplement: Supplementary file 1 — Supplementary Information. [file 41598_2020_60298_MOESM1_ESM.docx]

**A comparative analysis of link removal strategies in complex weighted networks**

Bellingeri M.^1*^, Bevacqua D.^2^, Scotognella F.^3,4^, Alfieri R.^1^, Cassi D.^1^

^1^Dipartimento di Fisica, Università di Parma, via G.P. Usberti, 7/a, 43124 Parma, Italy

* Corresponding author: [michele.bellingeri@unipr.it](mailto:michele.bellingeri@unipr.it)

^2^PSH, UR 1115, INRA, 84000, Avignon, France

^3^Dipartimento di Fisica, Istituto di Fotonica e Nanotecnologie CNR, Politecnico di Milano, Piazza Leonardo da Vinci 32, 20133 Milano, Italy

^4^Center for Nano Science and Technology@PoliMi, Istituto Italiano di Tecnologia, Via Giovanni Pascoli, 70/3, 20133, Milan, Italy

Supplementary materials

**S.1. The node transitivity**

**The binary node transitivity**

In network theory, a triangle is a triplet of nodes or subgraph of three nodes and the neighbors of a node are the node connected to that node^1^. The binary node transitivity (or clustering coefficient) is the ratio between the number of closed triangles among the neighbors of a node, and the total number of all possible triangles centered on that node. The transitivity of a node is also the proportion of links between the nodes within its neighbourhood divided by the number of links that could possibly exist between them.

The transitivity is defined:

$C_{i}=\text{ }\frac{\lambda_{i}}{\frac{1}{2}k_{i}(k_{i}-1)}$ (1)

where *λ_i_* is the number of closed triangles among neighbors of node i and $\frac{1}{2}k_{i}(k_{i}-1)$ is the total possible number of triangles centered on node i.

Defining $\tau_{i}=\frac{1}{2}k_{i}(k_{i}-1)$, we ca rewrite the node transitivity as:

$C_{i}=\text{ }\frac{\lambda_{i}}{\tau_{i}}$ (2)

**The weighted node transitivity**

There are several generalizations of transitivity to weighted graphs, here we use the definition by Barrat et al.^2^, this is a local vertex-level quantity, its formula is:

$C_{w}^{i}=\text{ }\frac{1}{s_{i}(k_{i}-1)}\sum_{j,h} \frac{w_{ij}+w_{ih}}{2}a_{ij}a_{ih}a_{jh}$ (3)

*s_i_* is the strength of vertex *i*, *a_ij_* are elements of the adjacency matrix, *k_i_* is the vertex degree, *w_ij_* are the weights.

**S.2. Additional results**


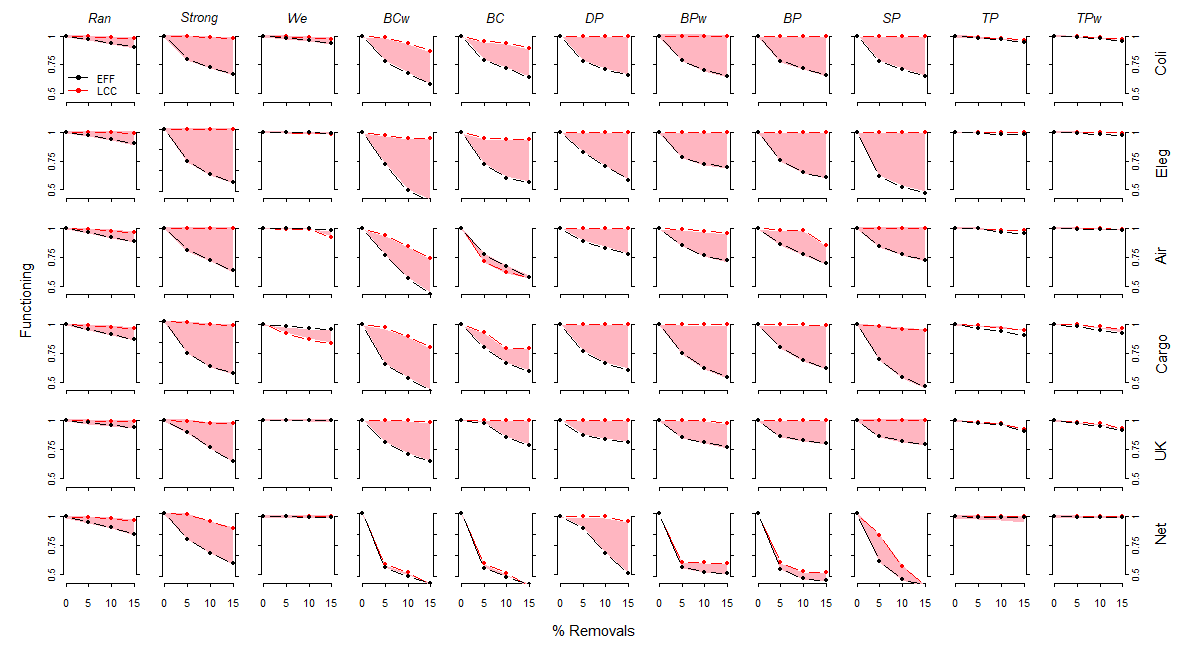


**Figure S1**: **Real-world complex weighted networks functioning decrease (*LCC* & *Eff*) under *q*= 5, 10, 15% of links removed**. The system functioning is normalized by the initial functioning value (e.g. before any removal). The pink area depicts the difference between *LCC* and *Eff* measures along the link removal process. Link removal strategies: random (*Ran*), strong (*Str*), weak (*We*), link weighted betwenness centrality (*BCw*), link binary betwenness centrality (*BC*), end nodes degree product (*DP*), end nodes betwenness centrality product (*BPw*), end nodes betwenness centrality product (*BPw*), end nodes strength product (*SP*), end nodes binary transitivity product (*TP*), end nodes weighted transitivity product (*TPw*).


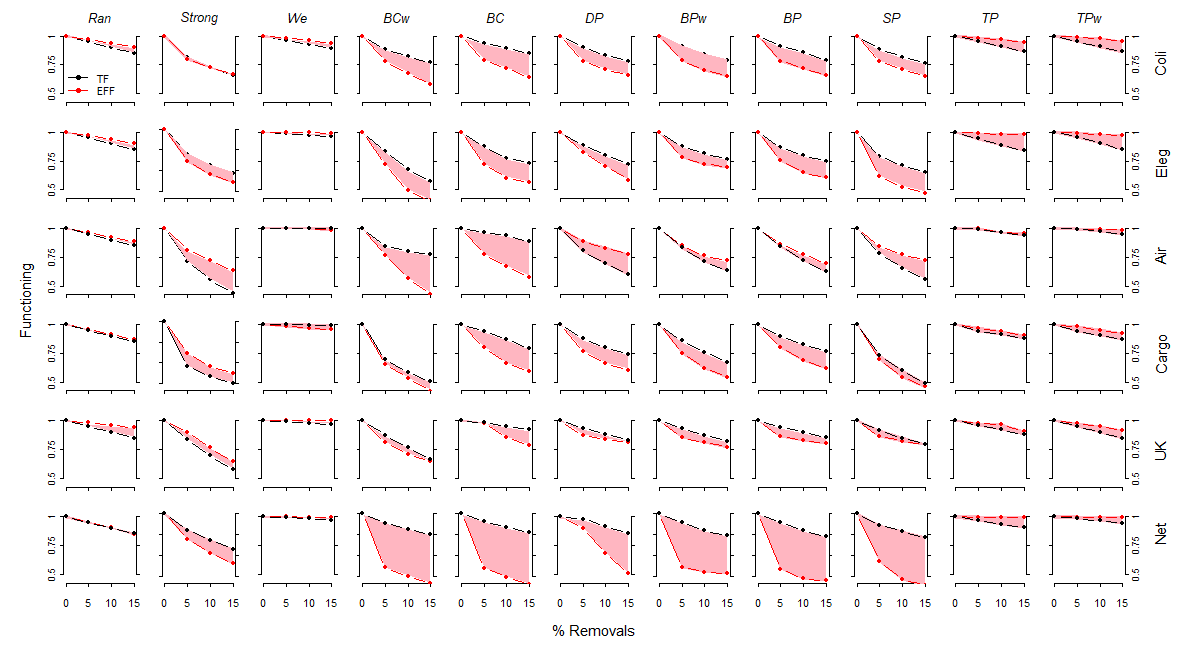


**Figure S2**: **Real-world complex weighted networks functioning decrease (*TF&* *Eff*) under *q*= 5, 10, 15% of links removed**. The system functioning is normalized by the initial functioning value (e.g. before any removal). The pink area depicts the difference between *TF* and *Eff* measures along the link removal process. Link removal strategies: random (*Ran*), strong (*Str*), weak (*We*), link weighted betwenness centrality (*BCw*), link binary betwenness centrality (*BC*), end nodes degree product (*DP*), end nodes betwenness centrality product (*BPw*), end nodes betwenness centrality product (*BPw*), end nodes strength product (*SP*), end nodes binary transitivity product (*TP*), end nodes weighted transitivity product (*TPw*).


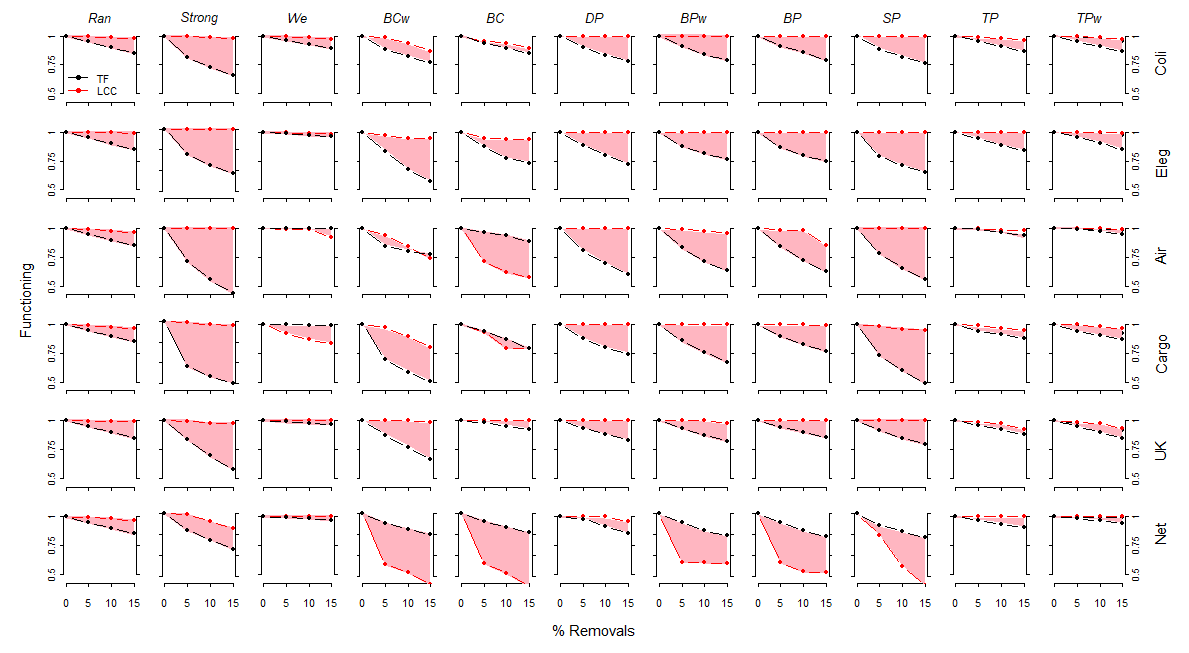


**Figure S3**: **Real-world complex weighted networks functioning decrease (*TF* & *LCC*) under *q*= 5, 10, 15% of links removed**. The system functioning is normalized by the initial functioning value (e.g. before any removal). The pink area depicts the difference between *TF* and *LCC* measures along the link removal process. Link removal strategies: random (*Ran*), strong (*Str*), weak (*We*), link weighted betwenness centrality (*BCw*), link binary betwenness centrality (*BC*), end nodes degree product (*DP*), end nodes betwenness centrality product (*BPw*), end nodes betwenness centrality product (*BPw*), end nodes strength product (*SP*), end nodes binary transitivity product (*TP*), end nodes weighted transitivity product (*TPw*).

**References**

1. Boccaletti S., Vito, L., Y., M., M, C. & D., H. Complex networks: Structure and dynamics. *Phys. Rep.* **424**, 175–308 (2006).

2. Barrat, A., Barthélemy, M., Pastor-Satorras, R. & Vespignani, A. The architecture of complex weighted networks. *Proc. Natl. Acad. Sci. U. S. A.* **101**, 3747–3752 (2004).
