# Supplementary material for: A comparative analysis of link removal strategies in real complex weighted networks
Source: Sci Rep. 2020 Mar 3;10:3911. doi: 10.1038/s41598-020-60298-7 (PMC7054356; doi:10.1038/s41598-020-60298-7)
Supplement: Supplementary file 2 — Supplementary Information2. [file 41598_2020_60298_MOESM2_ESM.docx]

**A comparative analysis of link removal approaches in complex weighted networks**

Bellingeri M.^1*^, Bevacqua D.^2^, Scotognella F.^3,4^, Cassi D.^1^

^1^Dipartimento di Fisica, Università di Parma, via G.P. Usberti, 7/a, 43124 Parma, Italy

* Corresponding author: [michele.bellingeri@unipr.it](mailto:michele.bellingeri@unipr.it)

^2^PSH, UR 1115, INRA, 84000, Avignon, France

^3^Dipartimento di Fisica, Istituto di Fotonica e Nanotecnologie CNR, Politecnico di Milano, Piazza Leonardo da Vinci 32, 20133 Milano, Italy

^4^Center for Nano Science and Technology@PoliMi, Istituto Italiano di Tecnologia, Via Giovanni Pascoli, 70/3, 20133, Milan, Italy

Supplementary materials

**S.1. Additional results**

**Figure S1**: **Real-world complex weighted networks functioning decrease (*LCC* & *EFF*) under *q*= 5, 10, 15% of links removed**. The system functioning is normalized by the initial functioning value (e.g. before any removal). The pink area depicts the difference between *LCC* and *EFF* measures along the link removal process. Link removal strategies: random (*Ran*), strong (*Str*), weak (*We*), link weighted betwenness centrality (*BCw*), link binary betwenness centrality (*BC*), end nodes end nodes degree product (*DP*), end nodes betwenness centrality product (*BPw*), end nodes betwenness centrality product (*BPw*), end nodes strength product (*SP*).

**Figure S2**: **Real-world complex weighted networks functioning decrease (*TF&* *EFF*) under *q*= 5, 10, 15% of links removed**. The system functioning is normalized by the initial functioning value (e.g. before any removal). The pink area depicts the difference between *TF* and *EFF* measures along the link removal process. Link removal strategies: random (*Ran*), strong (*Str*), weak (*We*), link weighted betwenness centrality (*BCw*), link binary betwenness centrality (*BC*), end nodes end nodes degree product (*DP*), end nodes betwenness centrality product (*BPw*), end nodes betwenness centrality product (*BPw*), end nodes strength product (*SP*).

**Figure S3**: **Real-world complex weighted networks functioning decrease (*TF* & *LCC*) under *q*= 5, 10, 15% of links removed**. The system functioning is normalized by the initial functioning value (e.g. before any removal). The pink area depicts the difference between *TF* and *LCC* measures along the link removal process. Link removal strategies: random (*Ran*), strong (*Str*), weak (*We*), link weighted betwenness centrality (*BCw*), link binary betwenness centrality (*BC*), end nodes end nodes degree product (*DP*), end nodes betwenness centrality product (*BPw*), end nodes betwenness centrality product (*BPw*), end nodes strength product (*SP*).
